# Acute Diffuse Peritonitis Due to Spontaneous Rupture of an Infected Endometrioma: A Case Report

**DOI:** 10.15388/Amed.2021.28.2.20

**Published:** 2021-12-22

**Authors:** Evelina Petruškevičiūtė, Diana Bužinskienė

**Affiliations:** Faculty of Medicine, Vilnius University, Vilnius, Lithuania; Clinic of Obstetrics and Gynaecology, Institute of Clinical Medicine, Faculty of Medicine, Vilnius University, Vilnius, Lithuania

**Keywords:** infected endometrioma, ovarian abscess, endometriotic ovarian cyst, complicated endometriosis

## Abstract

**Background.:**

Endometriosis is defined as a chronic, inflammatory, estrogen-dependent gynaecologic disease. It affects approximately 5–10% of reproductive-age women worldwide. Ovarian endometriosis is the most frequent form of this condition. Endometriotic cysts are found in about 17–44% of women diagnosed with endometriosis. It is well known, that ovarian endometriomas can cause infertility and chronic pelvic pain. Enlarging cysts can also cause ovarian torsion. In addition, ovarian endometriosis slightly increases the risk for ovarian cancer. The rupture of endometriotic ovarian cysts is an exceptional complication. According to the literature, the prevalence is less than 3% among women with endometriosis. The rupture of an ovarian endometrioma can cause acute peritonitis, which can lead to sepsis, septic shock and can be lethal. The occurrence of abscesses within an ovarian endometrioma is an extremely rare complication. Generally, the origin of infected endometriotic ovarian cysts is related to the previous invasive procedures involving pelvic organs or the use of intrauterine devices. Also, ovarian abscesses can be caused by the hematogenous or lymphatic spread of bacteria. Although, the literature points out that infection of endometriotic ovarian cysts can develop spontaneously. In these rare cases, reservoir and route of infection remains an enigma.

**Case report.:**

A 49-year-old female was brought to the emergency room with severe generalized lower abdominal pain (6/10) and three days lasting fever. Abdominal examination revealed diffuse abdominal pain with anterior abdominal wall muscle tension. Painful solid masses were felt on both sides of the uterus during the pelvic examination. Cystic masses were detected in both ovaries during transvaginal sonography. Computer tomography (CT) of the abdomen and pelvis revealed abnormal changes in both ovaries. A small amount of free fluid was found in the pelvic cavity along with thickened pelvic peritoneum. Suspecting acute peritonitis and bilateral tubo-ovarian abscesses, surgical treatment was performed. Lower midline laparotomy with bilateral adnexectomy and abdominal lavage with 4000 ml normal saline were done. The outcome of peritonitis was evaluated using the Mannheim peritonitis index (MPI=17 – low risk of morbidity and mortality). The histopathological examination revealed the diagnosis of bilateral endometriotic cysts complicated with acute inflammation, with associated acute inflammation of both fallopian tubes. Microbiological cultures from the purulent fluid were negative.

**Conclusions.:**

Although the occurrence of abscesses within an ovarian endometrioma is an extremely rare finding in clinical practice, it has to be considered by gynaecologists because it might result in a surgical emergency that can be life-threatening. Being aware of the risk factors of abscesses within an endometrioma can lead to an early diagnosis of this rare condition and help to avoid serious complications.

## Abstract (lt)

**Ūminis difuzinis pūlingas peritonitas, sukeltas plyšusių supūliavusių kiaušidžių endometrioidinių cistų. Klinikinio atvejo aprašymas:**

**Santrauka. *****Apžvalga. ***Endometriozė yra lėtinė, recidyvuojanti, uždegiminė ginekologinė liga, nustatoma bendrosios populiacijos 5–10 proc. reprodukcinio amžiaus moterų. Dažniausia endometriozės forma yra kiaušidžių endometrioma, diagnozuojama 17–44 proc. atvejų. Dažniausios endometriozės komplikacijos yra nevaisingumas ir lėtinis dubens skausmas. Didėjančios endometrioidinės cistos gali sukelti kiaušidės apsisukimą. Be to, sergant kiaušidžių endometrioze, didėja rizika išsivystyti kiaušidžių vėžiui. Kiaušidžių endometrioidinių cistų plyšimas yra reta komplikacija, nustatoma mažiau nei 3 proc. moterų, sergančių endometrioze. Ši komplikacija gali sukelti ūminį peritonitą, sepsį, septinį šoką ar net mirtį. Mokslinėje literatūroje minima dar viena reta komplikacija – absceso formavimasis endometriomoje. Dažniausiai infekcija endometrioidinėse cistose išsivysto dėl dubens srityje atliktų invazinių procedūrų arba dėl netinkamo intrauterinių prietaisų naudojimo. Taip pat abscesų susidarymą kiaušidėse gali sukelti į endometriomą hematogeniniu arba limfogeniniu keliu patekę mikroorganizmai iš kitų organizme esančių infekcijos židinių. Mokslinės literatūros šaltiniuose yra aprašyta keletas itin retų atvejų, kai abscesas kiaušidžių endometriomoje susiformavo spontaniškai, be žinomų rizikos veiksnių, nepavykus identifikuoti infekcijos šaltinio.

***Atvejo aprašymas. ***49 metų pacientė atvyko į skubios pagalbos skyrių dėl stipraus pilvo skausmo (6/10) ir tris dienas trunkančio subfebrilaus karščiavimo. Pilvo apčiuopos metu nustatytas viso pilvo ploto skausmingumas, jaučiami pilvaplėvės dirginimo ir priekinės pilvo sienos raumenų įtempimo simptomai. Taip pat abipus gimdos čiuopiami skausmingi, lygiu paviršiumi, vidutinio kietumo dariniai. Transvaginalinio ultragarsinio tyrimo metu dubenyje buvo matomi cistiniai dariniai abiejose kiaušidėse. Pilvo ir dubens organų kompiuterinėje tomografijoje rasta pokyčių abiejose kiaušidėse, taip pat buvo laisvo skysčio ir aiškiai infiltruota pilvaplėvė. Ginekologijos skyriuje pacientei skubos tvarka buvo atlikta apatinė išilginė laparotomija, kurios metu buvo rastas ūminis difuzinis pūlingas peritonitas ir abipusiai plyšę tuboovariniai abscesai, todėl pacientei buvo atlikta abipusė adneksektomija, kruopščiai išplauta pilvo ertmė. Operacijos metu pacientės būklė buvo įvertinta pagal Manheimo peritonito indeksą – nustatyta maža sergamumo ir mirtingumo rizika (MPI = 17). Pašalintos kiaušidės ir kiaušintakiai buvo nusiųsti histologiniam ištyrimui. Patohistologinio tyrimo metu buvo nustatytos abipusės endometrioidinės kiaušidžių cistos su ūminiu uždegimu ir ūminis abipusis kiaušintakių uždegimas. Mikrobiologiniame pasėlyje iš pūlingo pilvo ertmės skysčio bakterijų augimo nerasta.

***Išvados. ***Kiaušidžių endometriomos infekcija yra itin retai aptinkamas reiškinys klinikinėje gydytojų akušerių-ginekologų praktikoje, tačiau svarbu mokėti šią patologiją diferencijuoti nuo kitų ūminio pilvo sindromo klinikinius simptomus sukeliančių ligų. Laiku nediagnozuoti gimdos priedų abscesai gali plyšti ir sukelti pacientės gyvybei pavojingą ūminę būklę, sepsį ar net septinį šoką. Svarbu žinoti rizikos veiksnius, galinčius sukelti abscesų formavimąsi kiaušidžių endometrioidinėse cistose, nes tai gali padėti laiku diagnozuoti šią patologiją ir išvengti sunkių komplikacijų.

**Raktažodžiai: **infekuota endometrioma, kiaušidės abscesas, kiaušidės endometrioidinė cista, endometriozės komplikacijos

## Introduction

Endometriosis is defined as a chronic, inflammatory, estrogen-dependent gynaecologic disease [1]. It affects approximately 5–10% of reproductive-age women worldwide [2]. Ovarian endometriosis is the most frequent form of this condition. Endometriomas are found in about 17–44% of women diagnosed with endometriosis [1]. The typical symptoms of ovarian endometrial cysts include dysmenorrhea, dyspareunia, infertility and ovarian masses [3]. It is well known that ovarian endometriomas can cause infertility and chronic pelvic pain [4]. Enlarging cysts can also cause an ovarian torsion [5]. In addition, ovarian endometriosis slightly increases the risk for ovarian cancer [6]. The rupture of endometriotic ovarian cysts is an exceptional complication (less than 3%) but may cause acute peritonitis [7]. The occurrence of abscesses within an ovarian endometrioma is an extremely rare complication [8]. We present a case of a 49-year-old female who developed acute diffuse peritonitis due to spontaneous rupture of an infected endometrioma.

## The clinical case

A 49-year-old female, gravida 0, para 0, was brought to the emergency room with severe generalized lower abdominal pain (6/10) and three days lasting fever. The patient had a medical history of laparoscopic cholecystectomy and laparoscopic appendectomy. Also, she had an obstetric-gynaecological history of cervical dysplasia and conisation of the cervix before 4 years. 

On assessment, patient’s temperature was 38.8°C, she was hemodynamically stable. Abdominal examination revealed diffuse abdominal pain with anterior abdominal wall muscle tension. Painful solid masses were felt on both sides of the uterus during the pelvic examination. C-reactive protein was 266.4mg/l. 

**Figure 1.**
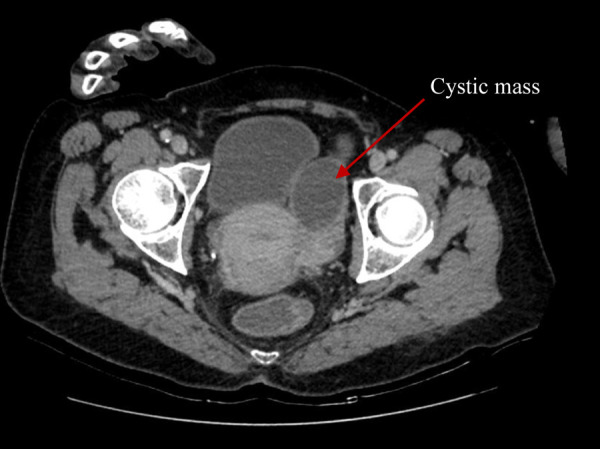
CT report of abdomen and pelvis. Cystic mass in the right adnexa of uterus (red arrow).

**Figure 2.**
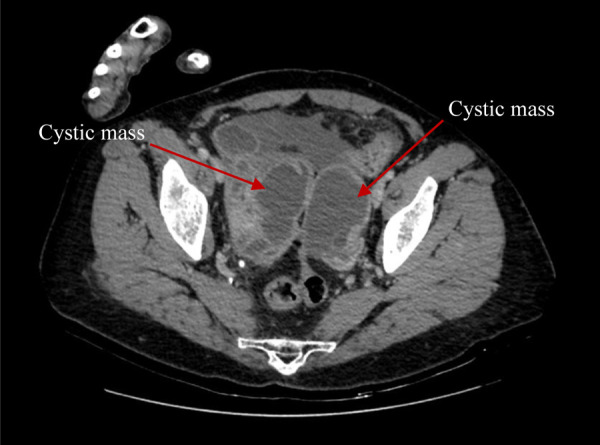
CT report of abdomen and pelvis. Cystic masses in both ovaries (red arrows).

Cystic masses, measuring 6.8 × 6.6 cm in the right adnexa and 7.8 × 5.1 cm in the left adnexa were detected during transvaginal sonography. Computer tomography (CT) of the abdomen and pelvis (Figure 1 and 2) demonstrated abnormal changes in both ovaries. Size of multiloculated cystic lesion in the right ovary was 6.8 × 6.6 cm and 7.8 × 5.1 cm in the left ovary. A small amount of free fluid was found in the pelvic cavity along with thickened pelvic peritoneum. 

Surgical treatment was recommended for the patient. In the presence of clinically significant acute diffuse peritonitis, large cystic masses in both ovaries, suspecting bilateral tubo-ovarian abscesses with a high probability of intraperitoneal adhesions (due to past surgeries), it was decided to perform laparotomy rather than laparoscopy. 

A lower midline laparotomy was performed. During laparotomy, about 100 ml of pus was found in the abdominal cavity. Microbiological samples were taken for cultures. Multiple adhesions were found between pelvic organs. The pouch of Douglas was obliterated and infiltrated as well as parametrium on both sides. Ruptured bilateral tubo-ovarian abscesses and acute diffuse peritonitis were detected. 

The abdominal surgeon was called to the operating room. Adhesions were dissected. Bilateral adnexectomy and abdominal lavage with 4000 ml normal saline were performed. The outcome of secondary peritonitis was evaluated using the Mannheim peritonitis index (MPI=17 – low risk of morbidity and mortality).

The histopathological examination revealed the diagnosis of bilateral endometriotic cysts complicated with acute inflammation and diffuse wall infiltration with macrophages, polymorphonuclears and fibrin, with associated acute inflammation of both fallopian tubes. Microbiological cultures from the purulent fluid were negative. The patient was treated with antibiotic therapy (Metronidazole 500mg IV three times daily and Amoxiclav 1.2g IV four times daily). Also, venous thromboembolism prophylaxis and infusion therapy were administered. 

The postoperative period went without complications. The patient was discharged after eight days of hospitalization.

## Discussion

Spontaneous occurrence of abscesses within an ovarian endometrioma is an extremely rare complication. Generally, the origin of infected endometriotic cysts is related to the previous invasive procedures involving pelvic organs such as caesarean section, laparotomic, laparoscopic or transvaginal hysterectomy [9]. Infection of an endometrioma may occur after transcutaneous oocyte retrieval during assisted reproductive technology (ART). The most commonly reported microorganisms in the clinical cases are coagulase-negative staphylococci, such as *S. aureus*, followed by group B streptococcus and *E. coli *[10]. Formation of tubo-ovarian abscesses (TOA) in patients with ovarian endometriosis has also been reported after hysteroscopy and percutaneous or transvaginal needle aspiration of endometriotic ovarian cysts [11]. Infection of an endometrioma can occur as a result of pelvic inflammatory disease (PID). PID is an ascending infection, in most cases caused by sexually transmitted microorganisms such as *Chlamydia trachomatis *and* Neisseria gonorrhoeae *[12,13]*.* Kavoussi and her group reported a case of a woman with TOA involving an endometrioma who had a diagnosis of bacterial vaginosis (BV) [14]. The literature points out, that bacterial vaginosis increases the risk of an ascending pelvic inflammatory disease due to imbalanced vaginal microflora. As a result, vaginal flora, including anaerobic organisms, *Gardnerella vaginalis* and *Mycoplasma hominis* and *genitalium* can cause various gynecological lesions such as endometritis, salpingitis oophoritis, or abscesses [14]. In the international literature, there have been reported some clinical cases with infected endometriotic cysts that had a medical history of previous infections, such as gastrointestinal infection due to *Helicobacter cinaedi* or *Salmonella* [15,16] and genital tract infections [17]. These examples support a theory that ovarian abscesses in endometriomas can be caused by the hematogenous or lymphatic spread of bacteria [18]. 

The patient in our case has no known history of oocyte retrieval during ATR, recent pelvic surgery, bacterial vaginosis, pelvic inflammatory disease or other infections. This data shows that infection of endometriotic ovarian cysts can develop without any known risk factor. 

According to the literature, there are different theories related to the spontaneous development of an abscess in the endometrioma. First, women with ovarian endometriosis are more vulnerable to infections due to an altered immune environment in the ectopic endometrial tissue [19]. Second, the wall of ovarian endometriotic cysts is thinner than normal ovarian epithelium and may be susceptible to bacterial invasion [20]. Third, the altered menstrual blood collected in the endometrioma might be a suitable culture medium for various pathogens [21]. 

Kubota et al*. *[22] suggested that ovarian endometriosis might be associated with higher morbidity of TOA. The incidence of tubo-ovarian abscesses was 2.3% in women with endometrioma and only 0.2% in women without endometrioma (p=0.001) [22]. Similar results were obtained by Chen M-J and his group after examining 6,228 patients (p<0.001) [21]. They found that women with endometriosis in stages III and IV were at a higher risk to develop tubo-ovarian abscesses than those who did not have endometriosis [20]. It was also highlighted that nulliparity increases the risk. The patient in our case was nulligravidous and nulliparous women, so there is a possible relation between spontaneous development of a tubo-ovarian abscess and nulliparity in this case. 

Our patient did not experience dysmenorrhea, dyspareunia or other symptoms associated with endometriosis, so it is unknown how long she has this condition. It is hard to detect the reservoir and route of infection because of the absence of growth of pathogenic organisms. Microbiological cultures from the purulent fluid could be negative due to previous antibiotic therapy. 

The rupture of endometriotic ovarian cysts is an exceptional complication. According to the literature, the prevalence is less than 3% among women with endometriosis [23]. This complication can develop spontaneously, there are cases reported during menstruation [24] and periovulatory stages [25], but it more often occurs during pregnancy, after abdominal and pelvic trauma [26]. Also, the risk is higher when the diameter of endometrioma is ≥6cm [27]. Takami et al. [26] reported a 2.8% incidence of the rupture of ovarian endometrioma during pregnancy. They also found enlarging cyst size, adhesion to the adjacent organs and the pressure of rapidly growing uterus being the main risk factors associated with rupture. Some authors suggested that this rare complication in pregnant patients is caused by the decidualization process, which results in an increased tension inside the endometriotic cyst [28]. According to the literature, ruptured ovarian endometriotic cysts can cause obstetrical emergencies such as acute hemoperitoneum [29] and ruptured uterine vessels [30], which may lead to the death of the fetus. These cases indicate that pregnant women with a history of endometriosis require closer attention.

Usually, patients with ruptured endometrioma present with acute abdominal pain, nausea, vomiting and fever, followed by peritoneal signs [31]. Transvaginal or abdominal ultrasound is necessary to assess the adnexal masses and to prove the diagnosis [32]. This complication has to be considered when free fluid and cystic ovarian lesions are detected. Magnetic resonance imaging (MRI) or computer tomography (CT) might be needed to make a definitive diagnosis if ultrasonography shows only nonspecific findings [33]. In our case, the rupture of an infected ovarian endometrioma caused acute diffuse peritonitis, and our patient experienced significant abdominal pain (6/10) and fever. Abdominal examination revealed peritoneal irritation and muscle rigidity. Transvaginal sonography and CT detected that both left and right ovarian lesions were large (≥6cm), which could have been a possible cause of the rupture.

In clinical practice, the serum level of cancer antigen (CA)-125 is used to distinguish benign and malignant ovarian tumours [34]. Usually, the amount of serum CA-125 in benign conditions is less than 200 U/ml [35]. In the international literature, there have been reported some clinical cases when ruptured ovarian endometrioma presented with exceedingly high CA-125 value, which imitated ovarian cancer [24,36,37]. Kurata et al. [24] concluded that not only CA-125 but CA19-9 as well significantly elevates in cases of ruptured endometriotic cysts (p=0.001). Tanaka and her group [38] obtained that quickly raised concentration of plasma D-dimer is linked with spontaneous rupture of ovarian endometrioma (p<0,001). These markers can be useful for clinicians to identify a ruptured endometriotic cyst diagnosis.

This case report alerts gynaecologists that the presence of infection in endometriomas can result in a surgical emergency if there is a rupture. According to the literature, diffuse peritonitis due to spontaneous rupture of tubo-ovarian abscesses can cause sepsis, septic shock and could be lethal [39]. Awareness of an acute clinical state and timely operative intervention are necessary for successful treatment.

## Limitations

Our case report does not reflect cases in the whole population. Also, our findings could have been influenced by other factors than those discussed in this report (coincidental factors).

## Conclusions

Although the occurrence of abscesses within an ovarian endometrioma is an infrequent finding in clinical practice, it has to be considered by gynaecologists, because it might result in a surgical emergency that can be life-threatening. Awareness of the risk factors of abscesses within an endometrioma can lead to an early diagnosis of this rare condition and help to avoid serious complications. In addition, pregnant patients with a history of endometriosis require closer attention and monitoring because the rupture of ovarian endometriotic cysts more often occurs during pregnancy and may cause obstetrical emergencies such as acute hemoperitoneum and ruptured uterine vessels.
